# Traveling into Outer Space: Unanswered Questions about Fungal Extracellular Vesicles

**DOI:** 10.1371/journal.ppat.1005240

**Published:** 2015-12-03

**Authors:** Marcio L. Rodrigues, Rodrigo M. C. Godinho, Daniel Zamith-Miranda, Leonardo Nimrichter

**Affiliations:** 1 Fundação Oswaldo Cruz (Fiocruz), Centro de Desenvolvimento Tecnológico em Saúde (CDTS), Rio de Janeiro, RJ, Brazil; 2 Instituto de Microbiologia Paulo de Góes, Universidade Federal do Rio de Janeiro, RJ, Brazil; Geisel School of Medicine at Dartmouth, UNITED STATES

## Introduction

**Fig 1:**
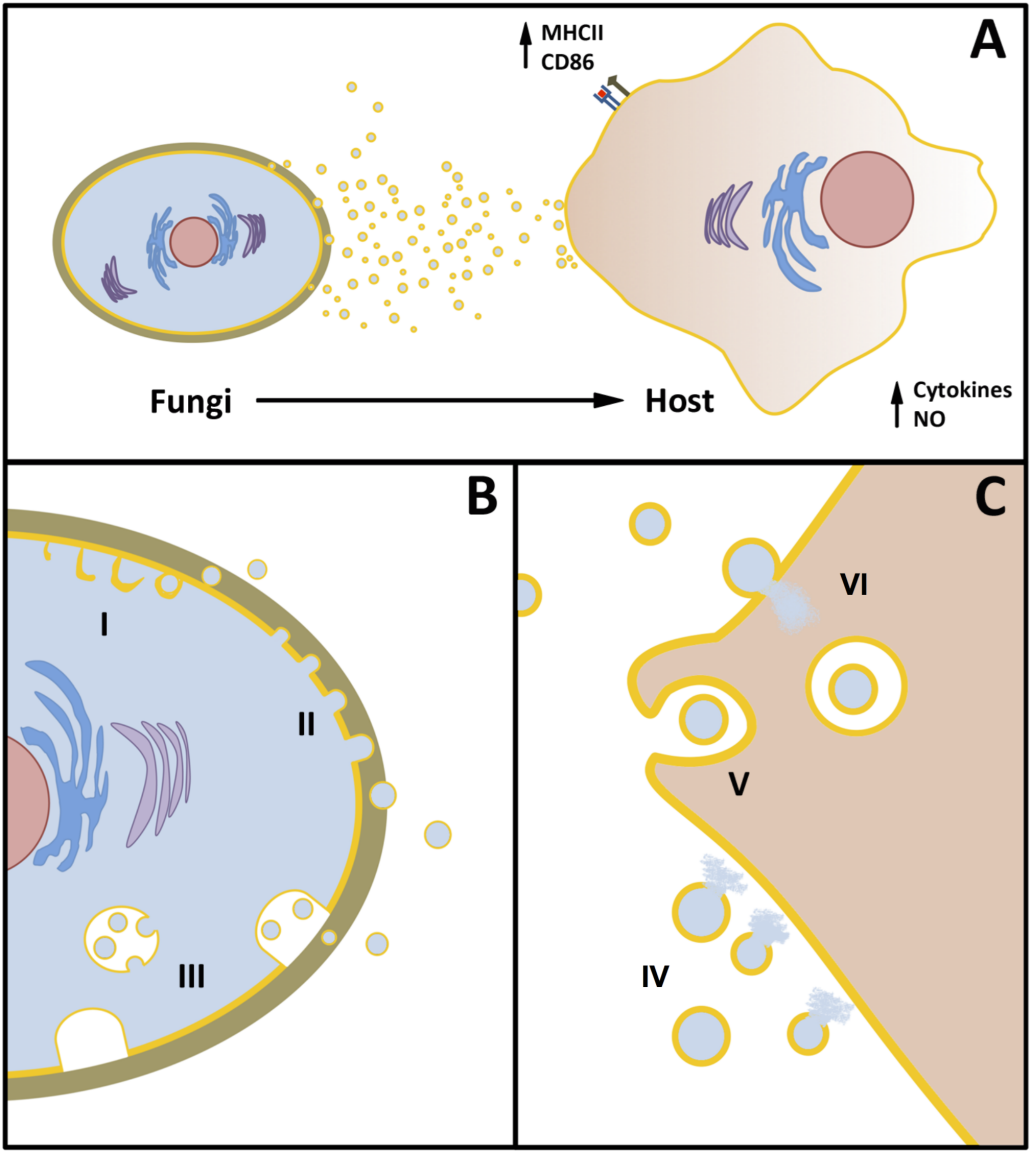
Overview of the functional aspects of fungal EVs. **A.** Fungal cells release heterogeneous populations of EVs that are immunologically active, as inferred from experimental models resulting in positive modulation of cytokine and nitric oxide (NO) production after exposure of host cells to EVs. Treatment of immune effector cells with EVs induces increased expression of CD86 and MHC-II molecules. For details and references, see Table 1. **B.** Biogenesis of fungal EVs is illustrated through (I) plasma membrane remodeling, resulting in cytoplasmic subtractions (inverted macropinocytosis), (II) membrane budding, resulting in ectosome formation, and (III) multivesicular body (MVB) formation, followed by fusion with the plasma membrane for the extracellular release of exosomes. **C**. The current literature supports the notion that fungal EVs can be lyzed for cargo release (IV). Alternatively, fungal EVs can be either internalized by (V) or fuse with the plasma membrane of host cells, likely resulting in the intracellular release of vesicular cargo (VI).

Extracellular vesicle (EV) release in fungi was described for the first time in 2007 in the yeast-like pathogen *Cryptococcus neoformans* [1]. Since then, the phenomenon of EV production, which is present in all domains of life, has been observed in many different fungal species, including yeast cells and hyphae. Composition of EVs, the impact of their release on fungal pathogenesis, and their potential use as protective immunogens have been explored in a number of original studies and comprehensive reviews (see Fig 1 and [2] for a summary). However, many aspects related to the biological properties of fungal EVs remain obscure. In this manuscript, we will focus our discussion on three fundamental but still unanswered questions about fungal EVs.

## What Is the Role of Fungal EVs during Infection?

It remains unknown whether fungal EVs are produced in vivo, which is likely linked to the lack of protocols and molecular markers for isolation of these membranous compartments from body fluids. Vesicle properties related to their stability in tissues are also obscure. *C*. *neoformans* EVs are rapidly disrupted by serum albumin at physiological concentrations [3]. This observation argues against the stability of EVs in vivo, but not against their potential functions. EV disruption might result in the release of internal and potentially immunomodulatory compounds into the extracellular space, possibly impacting the physiology of host cells.

Different studies provided indirect evidence supporting the hypothesis that fungal EVs are produced during infection. Sera from patients with cryptococcosis or histoplasmosis reacted with EV components [4,5]. In addition, EVs were isolated from plasma of patients with *Malassezia sympodialis*-associated atopic eczema [6]. In vivo studies with *C*. *neoformans* suggested that EVs are produced during lung infection [1].

The immunobiological activity of fungal EVs and the mechanisms by which they modulate host cell physiology have been first explored in *M*. *sympodialis*, where allergen-containing EVs induced IL-4 and TNF-α responses [6]. In further studies with *C*. *neoformans* and *Candida albicans* [7,8], labeling of EVs with DiIC_18_, a lipophilic and fluorescent stain, allowed observation of vesicle internalization by murine phagocytes and consequent cytokine production. EVs co-localized with the lipid raft marker GM1, suggesting the participation of such domains during vesicle internalization [7,8]. Apparently, fungal EVs are internalized through phagocytosis, since DiIC_18_ labeling was restricted to the cytoplasm after 15 minutes of incubation with phagocytes. It remains unclear, however, whether fungal EVs also fuse with host cell membranes, as suggested after interaction of *C*. *neoformans* EVs with human brain microvascular endothelial cells (HBMEC) [9]. In this situation, vesicle cargo would be directly delivered into the cytoplasm of host cells. This mechanism appears to modulate HBMEC permeability during murine cryptococcosis, facilitating crossing of the blood-brain barrier and brain colonization by the fungus [9]. More recently, Wolf and colleagues investigated a strain of *C*. *albicans* lacking expression of a phosphatidylserine synthase [10]. EV cargo lacked characteristic virulence factors, including phospholipase Plb3 and adhesin Sim1. These EVs failed to induce NFκB activation in macrophages [10]. Thus, phospholipid biosynthesis appears to be required for EV cargo and functions.

The molecules carried by EVs may impact antigen processing and, consequently, the immune response. *C*. *albicans* EVs stimulated dendritic cells (DCs) to produce IL-12p40, IL-10, and TNF-α, and induced upregulation of CD86 and MHC-II [7]. Treatment of murine macrophages with EVs from *C*. *neoformans* or *C*. *albicans* resulted in production of nitric oxide, IL-12, TGF-β, and IL-10 [7,8]. In addition, EVs from an acapsular strain of *C*. *neoformans* induced a high proinflammatory response [8]. The protective effect of EVs on the innate immune system has been suggested using the insect model *Galleria mellonella*. Treatment of larvae with EVs from *C*. *albicans* resulted in significant protection against subsequent challenges with this fungus [7]. These studies suggest that fungal EVs activate the innate immune response and may also promote, in other models, the development of adaptive responses (Table 1). A beneficial contribution of fungal EVs to humoral immunity is also expected. Enolase, HSP60, and GlcCer are examples of immunogens carried by EVs that can induce protective antibodies (reviewed in [11]).

**Table 1:** Functional diversity of fungal EVs.

Fungal pathogen	Reference	Induction of host cell responses	Influence on infection course
*C*. *neoformans*	[9]	Not determined	Enhanced traversal of the blood-brain barrier and infection of the central nervous system
*C*. *neoformans*	[8]	Tumor necrosis factor (TNF)-α, IL-10, transforming growth factor (TGF)-β, and nitric oxide (NO). Enhanced antifungal activity (macrophages).	Not determined
*Candida albicans*	[10]	Nuclear factor (NF) κB activation in macrophages	Not determined
*C*. *albicans*	[7]	NO, interleukin (IL)-12, TGF-β, and IL-10 (macrophages); IL-12p40, IL-10, and TNF-α (dendritic cells); up-regulation of CD86 and MHC-II	Protection in a *Galleria mellonella* model of infection
*Malassezia sympodialis*	[6]	IL-4 and TNF-α	Not determined

## EV Biogenesis: Where Do They Come From?

Exosomes and ectosomes are major EVs produced by eukaryotic cells. Exosomes consist of small (40–100 nm) vesicles originated by invagination of the endosomal compartments membrane, which is driven by a protein complex named endosomal sorting complex required for transport (ESCRT) [12]. This complex regulates the release of small vesicles inside the lumen of the endosome, generating the so-called multivesicular bodies (MVBs). Upon fusion with the plasma membrane, MVBs release exosomes as EVs to the outer space [12]. Unlike exosomes, ectosomes are larger (up to 1 μm), ubiquitous vesicles that are assembled at and released from the plasma membrane [13]. In fungi, mechanisms of vesicle biogenesis and extracellular release are still obscure. Therefore, these extracellular membranous compartments are still collectively called EVs.

MVB-like structures have been observed in *C*. *neoformans* [5]. *Saccharomyces cerevisiae* mutants lacking expression of ESCRT machinery proteins still produced EVs, but vesicle cargo was modified in the absence of ESCRT regulators [14]. Analysis of fungal EVs by electron microscopy revealed two kinds of populations in the cell wall periphery: large (up to 300 nm), individualized vesicles and small (up to 100 nm), grouped vesicles [15]. Groups of small EVs in the periplasm are consistent with exosome formation, as suggested in early studies with *C*. *neoformans* [16]. Observation of individualized and larger vesicles, however, is suggestive of membrane budding, likely resulting in ectosomes [17]. Membrane budding, in fact, has been observed more than a decade ago in *C*. *neoformans* [18]. EV formation can also include inverted macropinocytosis, a process by which fractions of the cytoplasm are sequestered by plasma membrane invaginations, resulting in individualized EV-like structures [19]. All the mechanisms described above would be consistent with the diversity in EV composition, which includes a number of cytoplasmic components (reviewed in [11]).

## Going Outside: How Do Fungal EVs Traverse the Cell Wall?

The conclusion that fungal EVs are originated either in the cytoplasm or at the plasma membrane levels implies that, to reach the outer space, passage through polysaccharide layers in the cell wall is required. In *C*. *neoformans*, three hypotheses are suggested to explain how fungal EVs traverse the cell wall [15], including (i) movement through channels, (ii) remodeling of the wall to facilitate EV transit, and (iii) mechanical pressure to force vesicle passage through small cell wall pores.

In contrast to plants, trans-cell wall channels have not been observed in fungal cells. In fact, in *C*. *neoformans*, vesicles interacted directly in the cell wall without any obvious trans-cell wall structures [1,15], which argues against the presence of a channel steering vesicles to the outer space. Atomic force microscopy studies demonstrated that fungal cell walls contain pores ranging from 1 to 400 nm [20,21], but visual evidence suggesting pore–EV association has not been provided so far, thus supporting cell wall remodeling as an effective mechanism of EV passage.

In *C*. *neoformans*, vesicles were observed next to areas of damaged cell walls [15], indicating that EVs could be released after cell wall injury. In this context, polysaccharide hydrolases associated with vesicles or with other cell wall components could play fundamental roles in cell wall passage. Compositional studies revealed the presence of glucan and/or chitin-degrading enzymes in EVs produced by *C*. *albicans*, *Paracoccidioides brasiliensis*, *Histoplasma capsulatum*, *S*. *cerevisiae*, and *C*. *neoformans* (reviewed in [11]). It is important to highlight, however, that the presence of EVs in association with damaged cell wall areas could alternatively denote mechanisms of cell wall repair, as well pointed out by Wolf and colleagues [15]. The detection of EV-associated enzymes with the ability to synthesize or modify cell wall polysaccharides in *S*. *cerevisiae* [14], *C*. *neoformans* [5], *P*. *brasiliensis*, [22] and *H*. *capsulatum* [4] is in agreement with this hypothesis. Cell wall remodeling and consequent EV release could be similar in other organisms. In *Staphylococcus aureus*, EVs are enriched with surface-associated or extracellular proteins, including N-acetylmuramoyl-l-alanine amidase [23], a cell wall hydrolase responsible for catalyzing cleavage of the bond between N-acetylmuramic acid and l-alanine in cell-wall glycopeptides. This enzyme was also detected in *Mycobacterium tuberculosis* vesicles [24]. In *Streptococcus pneumoniae*, EVs contained 1,4-β-N-acetylmuramidase [25], a peptidoglycan-degrading enzyme. Remarkably, these species and *M*. *tuberculosis* contained enzymes required for cell wall synthesis [24,25], suggesting that EV composition and cell wall remodeling are in fact associated. This supposition is in agreement with the observation that peptidoglycan-degrading enzymes create gaps within the peptidoglycan layer large enough to accommodate structures of high dimensions [26]. These independent observations may suggest that EV composition is responsible for the changes in microbial cell walls, resulting in vesicular passage through still-unknown mechanisms. The above-mentioned mechanisms of extracellular release would radically differ from those observed in mammalian cells and parasites. In these organisms, the plasma membrane is the most external layer surrounding the cells, implying no need for trans-cell wall passage [27,28]. Trans-cell wall passage is also unnecessary in Gram-negative bacteria. In these cells, EVs (namely outer membrane vesicles [OMVs]) correspond to spherical buds of the outer membrane filled with periplasmic content [29].

## Conclusions

Since the first isolation of fungal EVs in 2007, the progress made in the field was unquestionable. It is now clear that fungal EVs are part of a general mechanism of macromolecule export that results in the extracellular release of immunologically active components with the potential to modulate host responses either in favor of infection control or of fungal dissemination. Major questions, however, remain unanswered. The currently available literature clearly points to the need of improvement of protocols supporting the generation of knowledge on the role of fungal EVs in vivo, as well as on their biogenesis pathways and mechanisms by which they reach the outer space. The availability of sophisticated microscopy tools and well-established protocols for genetic manipulation in a number of fungal pathogens supports the notion that, although still long, the way to understanding the biological roles of fungal EVs can be shortened soon.
